# BEATS: BEAmline for synchrotron X-ray microTomography at SESAME

**DOI:** 10.1107/S1600577524005277

**Published:** 2024-07-15

**Authors:** Gianluca Iori, Mustafa Alzu’bi, Anas Abbadi, Yazeed Al Momani, Abdel Rahman Hasoneh, Pierre Van Vaerenbergh, Ivan Cudin, Jordi Marcos, Abdalla Ahmad, Anas Mohammad, Salman Matalgah, Ibrahim Foudeh, Mohammad Al Najdawi, Adel Amro, Abid Ur Rehman, Mohammad Abugharbiyeh, Rami Khrais, Amro Aljadaa, Mohammad Nour, Hussam Al Mohammad, Farouq Al Omari, Majeda Salama, María José García Fusté, Juan Reyes-Herrera, Christian Morawe, Maher Attal, Samira Kasaei, Charalambos Chrysostomou, Tomasz Kołodziej, Mateusz Boruchowski, Paweł Nowak, Jarosław Wiechecki, Anis Fatima, Andrea Ghigo, Adriana I. Wawrzyniak, Kirsi Lorentz, Giorgio Paolucci, Frank Lehner, Michael Krisch, Marco Stampanoni, Alexander Rack, Axel Kaprolat, Andrea Lausi

**Affiliations:** a SESAME – Synchrotron-light for Experimental Science and Applications in the Middle East, Allan, Jordan; b ESRF – The European Synchrotron, Grenoble, France; c Elettra-Sincrotrone Trieste SCpA, Basovizza, Trieste, Italy; d ALBA Synchrotron Cerdanyola Catalonia Spain; e The Cyprus Institute Nicosia Cyprus; f Solaris National Synchrotron Radiation Centre Jagiellonian University Krakow Poland; g Laboratori Nazionali di Frascati dell’INFN INFN Frascati Rome Italy; h Deutsches Elektronen-Synchrotron DESY Hamburg Germany; i Swiss Light Source Paul Scherrer Institut Villigen Switzerland; Advanced Photon Source, USA

**Keywords:** X-ray imaging, computed tomography, X-ray phase-contrast, microscopy, radiography, synchrotron radiation, cultural heritage, materials science, biomedical imaging

## Abstract

The ID10 beamline of the SESAME (Synchrotron-light for Experimental Science and Applications in the Middle East) synchrotron light source in Jordan was inaugurated in June 2023 and is now open to scientific users. The beamline, which was designed and installed within the European Horizon 2020 project BEAmline for Tomography at SESAME (BEATS), provides full-field X-ray radiography and microtomography imaging with monochromatic or polychromatic X-rays up to photon energies of 100 keV. The photon source generated by a 2.9 T wavelength shifter with variable gap, and a double-multilayer monochromator system allow versatile application for experiments requiring either an X-ray beam with high intensity and flux, and/or a partially spatial coherent beam for phase-contrast applications. Sample manipulation and X-ray detection systems are designed to allow scanning samples with different size, weight and material, providing image voxel sizes from 13 µm down to 0.33 µm. A state-of-the-art computing infrastructure for data collection, three-dimensional (3D) image reconstruction and data analysis allows the visualization and exploration of results online within a few seconds from the completion of a scan. Insights from 3D X-ray imaging are key to the investigation of specimens from archaeology and cultural heritage, biology and health sciences, materials science and engineering, earth, environmental sciences and more. Microtomography scans and preliminary results obtained at the beamline demonstrate that the new beamline ID10-BEATS expands significantly the range of scientific applications that can be targeted at SESAME.

## Abstract 2

The recently inaugurated beamline ID10-BEATS for hard X-ray full-field tomography at the SESAME synchrotron in Jordan is presented. The design, performance and scientific applications of the beamline, which was developed within the European Horizon 2020 project BEAmline for Tomography at SESAME, are illustrated.

## 1. Introduction

X-ray tomography is a widely non-destructive three-dimensional (3D) imaging technique which generates volume images of a specimen by using penetrating radiation. The method involves the acquisition of multiple projection images (radiographs) of angular views covering 180° or 360° of an object, and a mathematical procedure of 3D tomographic reconstruction. Its scientific and technological applications are vast, ranging from life and materials science to archaeology as well as earth and environmental research.

The application of synchrotron X-ray computed tomography (SXCT) started in the 1980s and is growing steadily, driven by the increase in brilliance of the X-ray sources, and by significant improvements in detector and computing technology. When compared to its laboratory counterpart, the advantages of SXCT are related to the high photon flux delivered on a small area of the sample, and to the possibility to extend the distance to the X-ray source to tens or even hundreds of meters (Withers *et al.*, 2021 ▸). The high photon flux enables on one hand short acquisition time and time-resolved scans (Rack *et al.*, 2013 ▸); on the other hand it allows tuning of the impinging X-ray energy with the use of monochromator optics, which provides images of superior contrast and quality. The large distance to the light source minimizes the effect of the finite source size on image formation. Radiographs obtained with parallel and partially coherent X-ray beams can achieve higher spatial resolution. Image contrast can be either generated via the difference in the photo-electric absorption (absorption-contrast) or electron density (phase-contrast) of the elements constituting the sample. Phase-contrast SXCT scans provide a sensitivity to light elements that is two to three orders of magnitude larger than that of absorption-contrast computed tomography (CT) in the hard X-ray regime (Cloetens *et al.*, 1996 ▸).

The project Beamline for Tomography at SESAME (BEATS) was funded by the EU via the H2020 programme, and brought together a consortium of research facilities in the Middle East (SESAME, Jordan and the Cyprus Institute, Cyprus), and European synchrotron radiation facilities and high-energy laboratories [ALBA, Spain; DESY, Germany; Elettra, Italy; European Synchrotron Radiation Facility (ESRF), France; INFN, Italy; Paul Scherrer Institut (PSI), Switzerland; and SOLARIS, Poland] with the goal to design, install and commission a beamline for hard X-ray full-field tomography at SESAME, fostering in this way the user community of the facility. The project was coordinated by the ESRF.

During the instrumentation design phase, several successful examples from established SXCT stations worldwide were studied, such as TOMCAT of the Swiss Light Source (PSI) (Stampanoni *et al.*, 2007 ▸), ID17 (Mittone *et al.*, 2020 ▸) and ID19 (Weitkamp *et al.*, 2010 ▸) of the ESRF, FaXToR of ALBA (Mittone *et al.*, 2022 ▸), SYRMEP of Elettra (Tromba *et al.*, 2010 ▸), TopoTomo of ANKA (now KARA, Germany) (Rack *et al.*, 2009 ▸), the Micro-Computed Tomography beamline of the Australian Synchrotron (Arhatari *et al.*, 2023 ▸), and the BAMline of BESSY-II (Germany) (Rack *et al.*, 2008 ▸).

This communication describes the instrumentation and performance of BEATS, which is fully commissioned for scientific user operation. First SXCT scans performed at the beamline are presented, demonstrating the huge scientific potential of the instrument for a broad range of disciplines.

## 2. Beamline design and instrumentation

**Figure 1:**
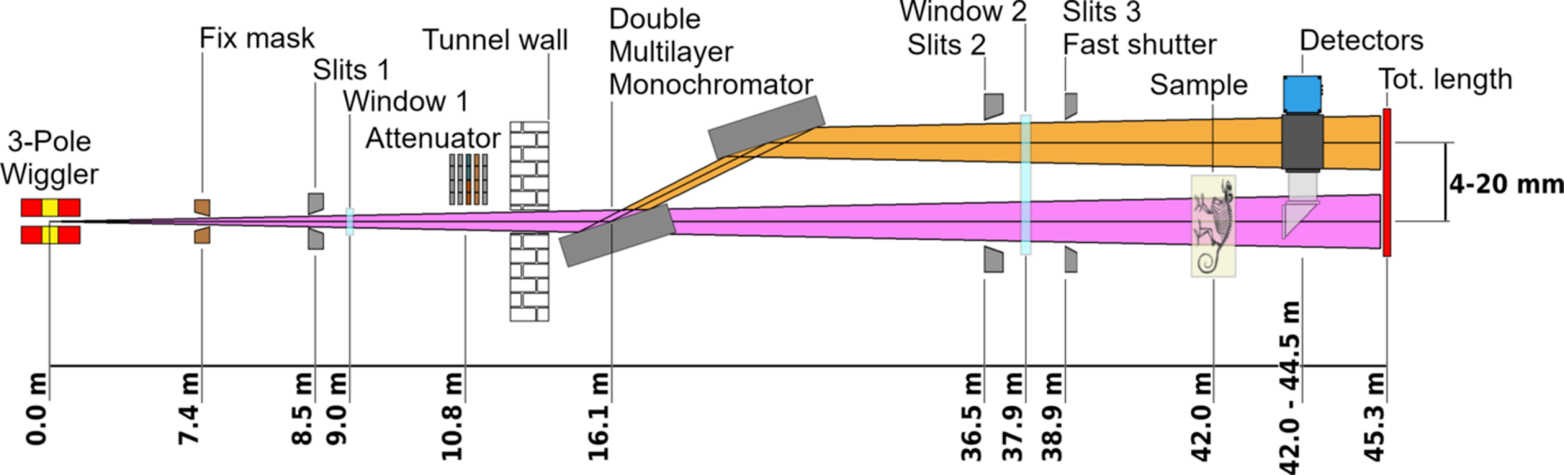
Schematic layout of the ID10-BEATS beamline of SESAME. Only optical and beam-defining elements are shown.

The beamline is composed of an insertion device (ID) and a front-end installed inside the synchrotron accelerator tunnel, and of two radiation shielded hutches connected by a vacuum transfer line, one hosting the beamline optics and the other the experimental endstation, respectively. An illustration of the beamline layout is shown in Fig. 1 ▸. The design is inspired by the TOMCAT beamline of the SLS (Switzerland) (Stampanoni *et al.*, 2007 ▸), and was validated by ray-tracing numerical simulation (Iori *et al.*, 2021*a* ▸).

### 2.1. X-ray source: three-pole wiggler

**Figure 2:**
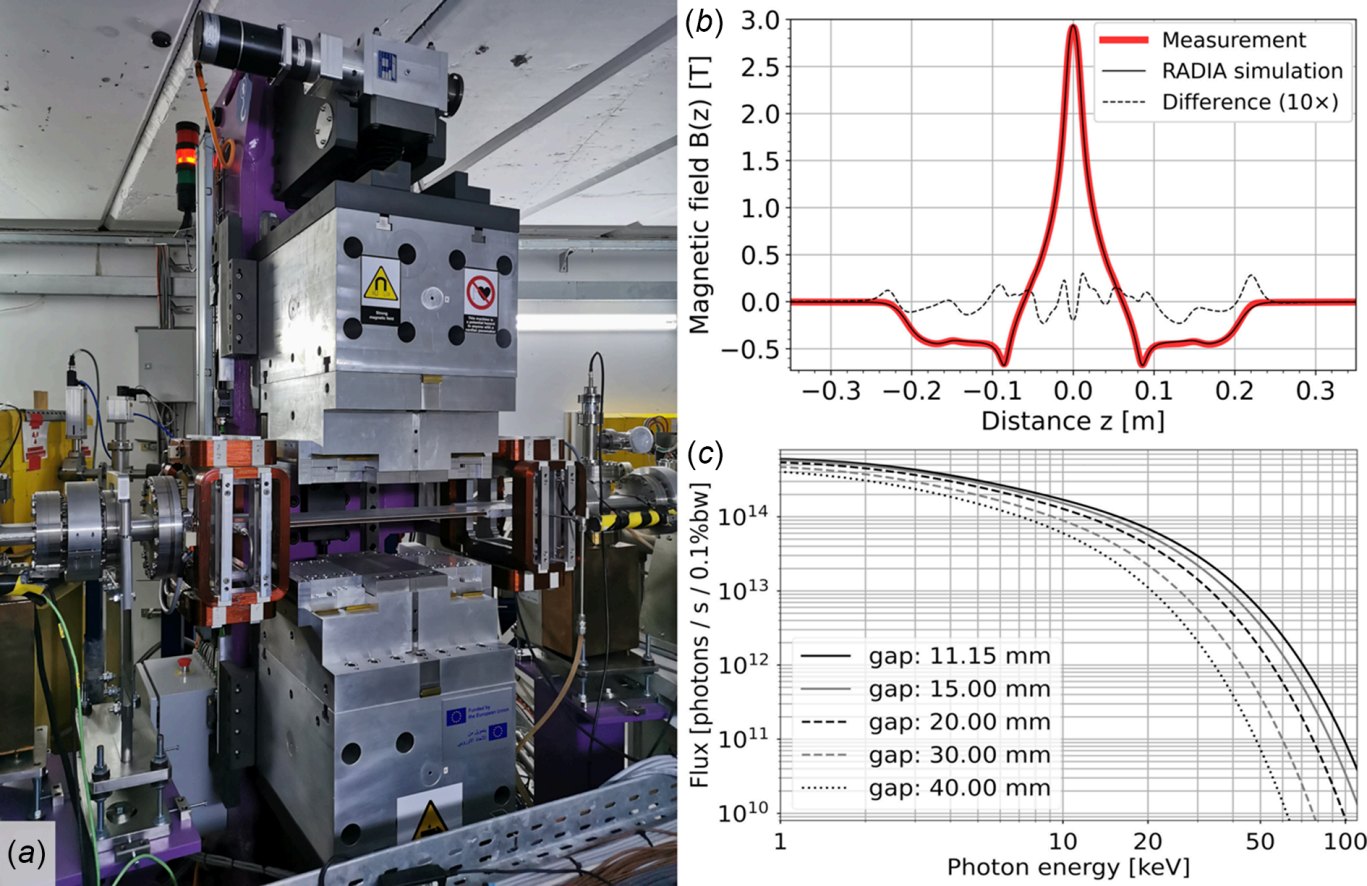
(*a*) BEATS three-pole wiggler insertion device installed in the SESAME storage ring. (*b*) Simulated and measured profiles of the vertical component of the magnetic field along the longitudinal axis of the ID, at minimum gap of 11.15 mm. Simulations were performed in *RADIA* (Elleaume *et al.*, 1997 ▸). (*c*) X-ray photon flux emitted by the BEATS 3PW at different magnetic gaps.

The BEATS photon source is a three-pole wiggler ID [3PW, Fig. 2 ▸(*a*)] with a central magnetic pole reaching 2.9 T peak magnetic field at minimum gap, installed in the short straight section of cell 10 of SESAME’s 2.5 GeV storage ring. The conceptual magnetic model of the ID was established in collaboration between ALBA (Spain) and the INFN (Italy) with the following objectives (Campmany *et al.*, 2021 ▸):

(i) Provide an X-ray point source with broadband energy spectrum and photon flux at the sample position of at least 1 × 10^10^ photons mm^−2^ s^−1^ at 50 keV.

(ii) Minimize the multipolar effects on the SESAME storage ring optics.

(iii) Reduce the attractive forces between the magnetic structures leading to minor mechanical constraints.

The main parameters of the ID are listed in Table 1 ▸. Plots of the magnetic field (simulated and measured) along the longitudinal axis of the 3PW device at the minimum gap of 11.15 mm are shown in Fig. 2 ▸(*b*). The energy spectrum of the emitted photon flux [Fig. 2 ▸(*c*)] reaches 100 keV, and can be tuned by adjustment of the ID gap before each experiment. Active correction coils [visible on both sides of the main magnetic structure in Fig. 2 ▸(*a*)] are operated in correlation with the gap setting to correct deviations of the electron beam trajectory.

### 2.2. Front-end

The beamline front-end comprises:

(i) A fixed mask defining a useful beamline aperture of 1.8 mrad  ×  0.36 mrad (H  ×  V).

(ii) Motorized, in-vacuum slits, used to adjust the beam size and reduce the heat load on downstream components.

(iii) A 0.5 mm-thick chemical vapour deposition (CVD) diamond window separating accelerator and beamline vacuum sections.

(iv) A white beam attenuator system composed of five motorized actuators with four cooled filters each.

(v) Radiation safety and vacuum shutters designed to absorb the beam heat load and high-energy X-rays, and to protect from contamination of the storage ring vacuum environment in case of outgassing or leak.

Available X-ray filters range from 5 mm glassy carbon (HTW, Germany) to polished plates of high-*Z* metals (0.5 mm W and Au), and can be used in succession to tune the intensity and average energy of the white beam, and to modulate the heat load on the first mirror of the monochromator. The front-end components were manufactured by JJ X-ray A/S (Denmark).

### 2.3. Double-multilayer monochromator

**Figure 3:**
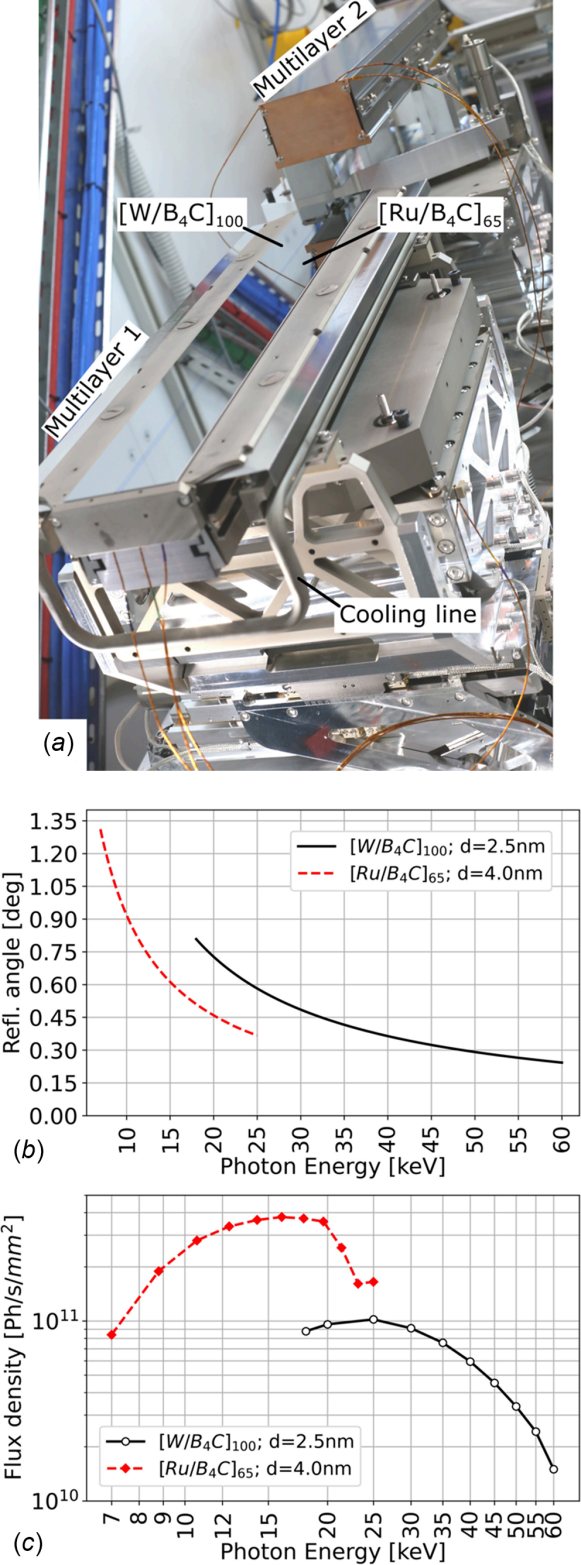
(*a*) Photograph of the BEATS DMM installed in the beamline’s optics hutch. The two multilayer coatings and the cooling circuit of the first optical element are indicated. (*b*) Reflection angle for both DMM stripes at different working energies. The reflection angle was calculated using the refraction corrected Bragg equation following Morawe (2019 ▸) and validated experimentally by scans of metal foils at the respective *K*-edge absorption energies. (*c*) Simulated monochromatic flux density at the sample position (42 m from source) obtained with the *XOPPY* (Sanchez del Rio & Dejus, 2004 ▸) and *ShadowOui* (Rebuffi & Sánchez del Río, 2016 ▸) tools contained in the *OASYS* suite (Rebuffi & Rio, 2017 ▸). Optical surfaces and multilayer properties were modelled using the surface metrology results provided by the substrate’s supplier and obtained at the ESRF Multilayer Laboratory (France), respectively.

The main beamline optical component is a vertical-reflecting double-multilayer monochromator [DMM, Fig. 3 ▸(*a*)] installed in a dedicated optics hutch at 16.1 m from the photon source. The device is used for applications requiring high X-ray energy sensitivity, phase-contrast scans of light or moderately absorbing materials, and for absorption edge subtraction imaging. Each optical element [multilayers 1 and 2 in Fig. 3 ▸(*a*)] is installed on its own set of remotely controlled positioners, avoiding any mechanical link and software cross-talk between the two multilayers. Three motorized axes allow the selection of the desired multilayer coating, and control over the reflection angle (multilayer pitch or Bragg angle) and beam offset. At the same time, both multilayers can be retracted from the beam path, allowing operation in white beam mode. The maximum power impinging on the first multilayer mirror (132.7 W) is dissipated through a gallium–indium eutectic alloy bath that also decouples mechanically the optical element from the water cooling line. The second multilayer positioner is equipped with a motorized roll stage, and a piezoelectric actuator in series with the stepper motor for fine-tuning of the reflection angle and therefore of the exit beam direction. Key figures of the DMM mechanical performance as measured at the manufacturer’s premises (Strumenti Scientifici CINEL, Vigonza, Italy) are reported in Table 2 ▸. During all measurements, suitable dummies of the multilayers were installed, replicating the shape and mass of the real optical elements. The resolution and repeatability of the pitch stages were measured in air with a laser interferometer (XL80, Renishaw, UK). Measurements of the natural frequencies of the pitch adjustment of each multilayer were carried out under vacuum driving the linear piezoelectric actuator of the pitch adjustment of multilayer 2 with a sinusoidal, fixed amplitude input signal. A frequency sweep in the range between 1 and 1000 Hz was performed in 1 Hz steps, simultaneously acquiring the encoder readout of each multilayer pitch stage in real time. The frequencies of the first two eigenmodes (Table 2 ▸) were detected from the Bode diagrams of the response of each pitch adjustment. The long-term vibration stability of each multilayer pitch was characterized as the r.m.s. value of its calibrated encoder readout over a period of 20 min, with the DMM under vacuum and no external heat or vibration sources in the surrounding area. Real-time data collection for vibration and stability analyses was performed using a beam enhanced stabilization technology (BEST) module manufactured by CAEN ELS (Italy). Two pairs of multilayer stripes (specifications in Table 2 ▸) were deposited on each Si substrate at the ESRF Multilayer Laboratory (France), varying the *d*-spacing along the multilayer longitudinal axis as described by Morawe (2007 ▸). The effect of the longitudinal substrate slope error was investigated by Iori *et al.* (2021*a* ▸). Energies between 7 and 60 keV can be selected by setting the reflection angle as shown in Fig. 3 ▸(*b*), with an energy resolution of 1.6% or 2.4% for stripe 1 and 2, respectively (Table 2 ▸). The photon flux density at the sample position when using the DMM is plotted for different photon energies in Fig. 3 ▸(*c*). 500 mm-long optical surfaces allow the usable beam height to be preserved even when working at very low reflection angles.

### 2.4. X-ray imaging endstation

**Figure 4:**
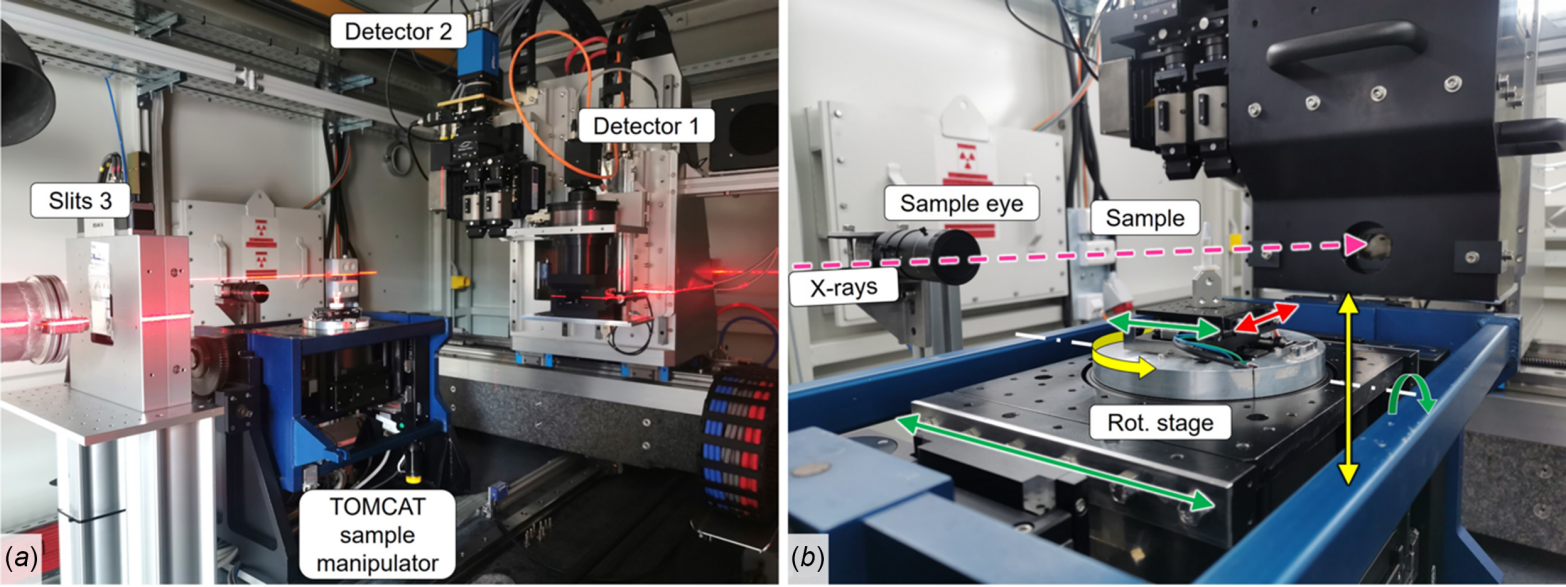
(*a*) BEATS X-ray radiography and tomography endstation with detectors 1 and 2 for white beam applications installed. For monochromatic beam experiments, detector 3 of Table 3 ▸ can be installed by replacing detector 2. A laser line helps the user in finding a preliminary sample alignment position. (*b*) Detail of the tomography sample manipulator indicating the axes of sample motion.

The imaging setup is hosted in a 9 m-long lead-shielded experimental hutch reaching 45.3 m from the photon source. The hutch hosts in-vacuum and sample (in-air) slits, a CVD diamond window separating vacuum and air environments, a fast shutter with minimum exposure time of 50 ms and 10 Hz repetition rate, used to limit the X-ray exposure of delicate samples (Muñoz Pequeño *et al.*, 2021 ▸), and the imaging endstation shown in Fig. 4 ▸(*a*), hosting manipulators for sample and detectors.

#### 2.4.1. Sample manipulator

The six-axis tomography sample manipulator of the TOMCAT beamline was donated to SESAME by the SLS (Stampanoni *et al.*, 2007 ▸). This is equipped with a high-precision air-bearing rotation stage [Fig. 4 ▸(*b*)] for payloads up to 5 kg, and an electrical feedthrough for the connection of sample environments. The propagation distance between sample and detectors can be adjusted between 0 and 2500 mm. A second sample endstation for payloads up to 50 kg and longer propagation distances will be commissioned in the second half of 2024. See Section 6 for more details on the future endstation and sample environment upgrades.

#### 2.4.2. Detectors

Tables 3 ▸ and 4 ▸ show the available detectors and cameras, respectively. Two full-field detectors can be installed on a common stage [Fig. 4 ▸(*a*)], which is designed to reduce the effect of floor vibrations on scan resolution (Mokoena *et al.*, 2023 ▸). Both cameras of Table 4 ▸ can be used interchangeably on all detectors of Table 3 ▸. All imaging systems are based on the indirect illumination principle described by Bonse & Busch (1996 ▸). The imaging systems based on microscope optics (detectors 1 and 3 in Table 3 ▸) and tandem macro photographic lenses (detector 2 in Table 3 ▸) are described by Douissard *et al.* (2012 ▸) and Mittone *et al.* (2017 ▸), respectively. Motorized stages responsible for detector positioning and change of magnification are equipped with absolute encoders. This allows overview scans to be performed followed by local zoom tomography, maintaining the control of the scan region of interest.

## 3. Data acquisition, processing and storage

### 3.1. Data acquisition system

**Figure 5:**
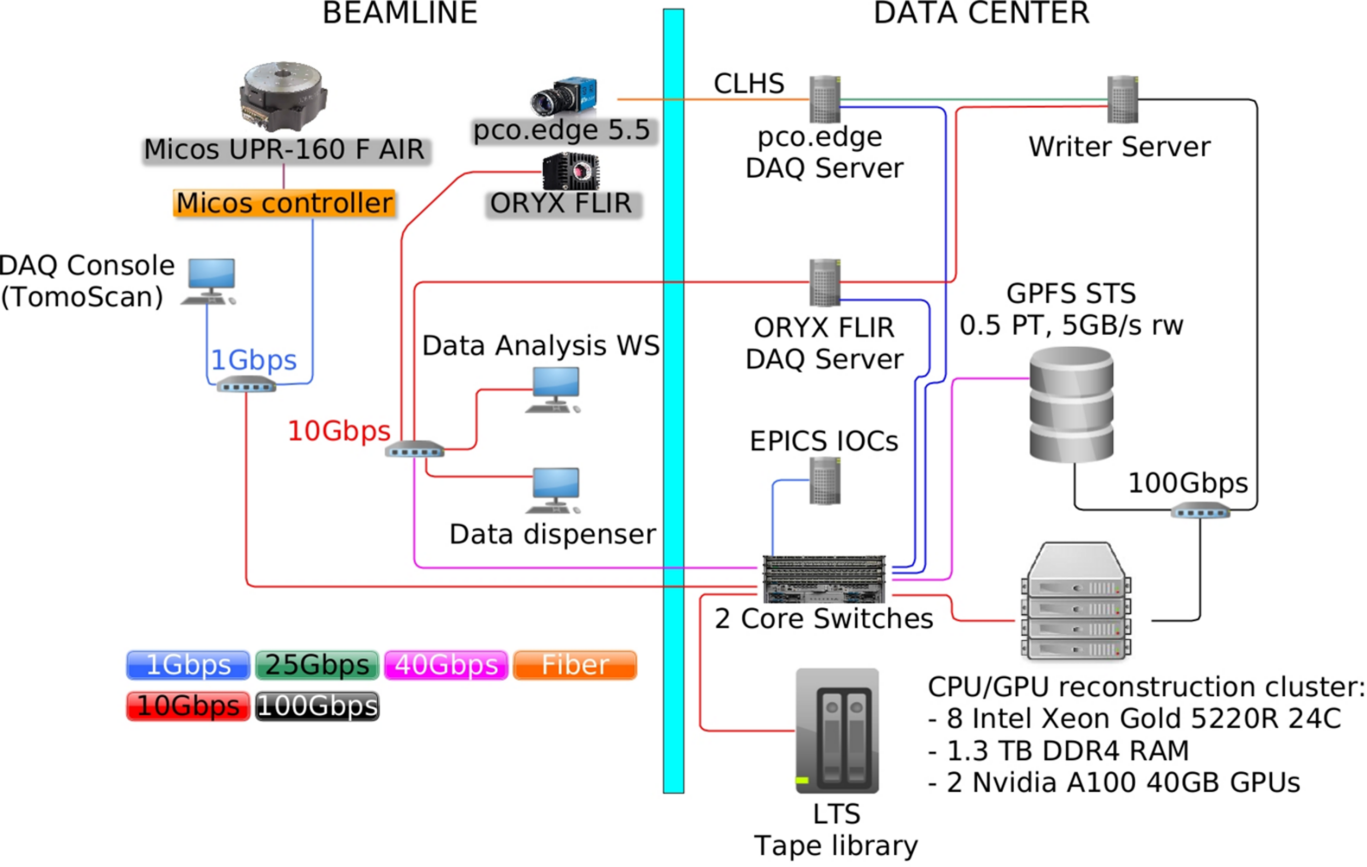
Layout of BEATS data acquisition, processing and storage infrastructure. Additional information is provided by Iori *et al.* (2021*b* ▸).

The BEATS data acquisition, processing and storage infrastructure is illustrated in Fig. 5 ▸ and described in a separate communication (Iori *et al.*, 2021*b* ▸). The system can handle a sustained detector data throughput of 8.8 Gb s^−1^, and was designed in collaboration with PSI and ESRF. All beamline experimental data are stored on a centralized 0.5 petabyte General Parallel File System (GPFS) short-term storage (STS in Fig. 5 ▸), and periodically archived to a magnetic tape long-term storage (LTS) facility in compliance with SESAME’s experimental data policy (Alzubi *et al.*, 2023 ▸). CT reconstructions are scheduled on a hybrid CPU/GPU cluster.

#### 3.1.1. Scan modalities

**Figure 6:**
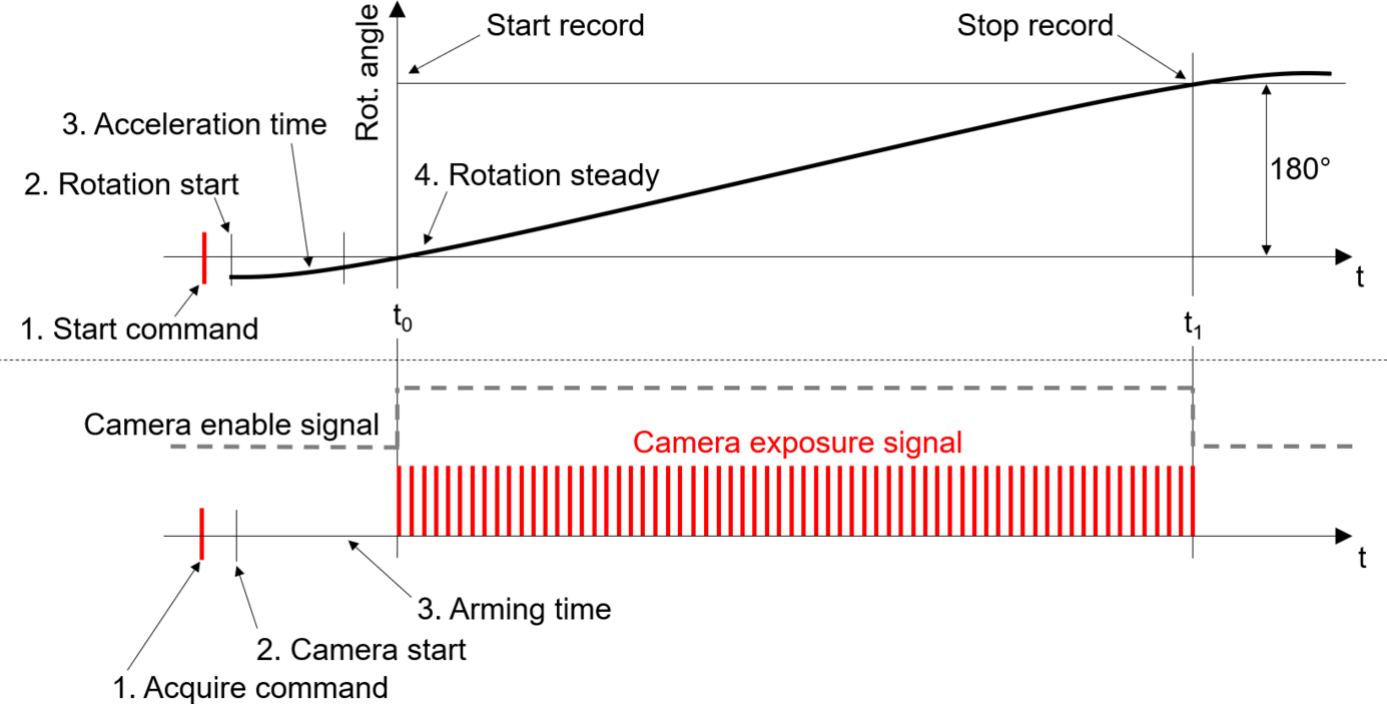
Implementation of a software-based continuous CT scan. The rotary stage acceleration and the camera arming time are compensated by initiating the sample rotation and camera frame collection processes ahead of the target start scan position. This approach ensures that, when capturing the first frame of the dataset, the rotary stage is moving at a steady speed.

Different data collection modalities are provided to users:

(i) Continuous scan: the sample is rotated with constant speed, while the camera continuously acquires projections, as illustrated in Fig. 6 ▸. While setting up the scan, the user can define the exposure time within the range allowed by the camera (Table 4 ▸). The frame time (time required for the collection of one image frame, limited by the maximum frame rate of the camera) is retrieved from the camera driver before launching the scan, and used to calculate the sample rotation speed. Exposure and frame time are in this way decoupled, allowing the exposure of the detector during a portion of the frame time. A buffer acceleration time is considered at the start of the scan. At the end of each scan, an array of estimated angular positions is calculated from the total number of projections, the speed of the rotation axis and the camera frame time. If the readout from the rotation stage encoder is available, an array of readout angular positions corresponding to each image frame is also stored. The continuous scan is used as the standard scan modality for tomography experiments.

(ii) Step scan: the rotation axis is moved and stopped at equidistant angular values to record projection frames. This is a slow scan mode that allows extended exposure time for each frame or averaging of multiple frames, and helps to suppress artefacts generated by sample rotation during the scan.

(iii) Single radiograph: the fast exposure shutter is controlled in combination with the camera shutter, and is closed during waiting periods in which the camera is not collecting frames (*e.g.* during alignment procedures). This modality is used during sample alignment, and to minimize X-ray exposure when collecting radiographs of delicate objects.

#### 3.1.2. Step scan triggering system

During step scans, the collection of individual frames must be triggered once the rotary stage has reached the required position. With the ORYX FLIR camera available at the beamline, this is done by specifying the total number of frames, setting the camera’s image mode to *multiple*, and by sending software-based triggers from the *TomoScan* application to the camera driver. For the pco.edge 5.5 camera, the combination of *multiple* frame collection and *software-trigger* is not available. Instead, single frames can be collected by setting the image mode to *single*, and by repeatedly sending *start acquire* commands. Nevertheless, this camera trigger modality involves approximately one second of additional arming time for each *start acquire* command. To overcome this issue, an external triggering server was designed and developed at SESAME, consisting of two main parts: (i) a hardware controller based on Raspberry Pi that can transmit trigger signals over extended cable lengths, and (ii) a software component programmed in C implementing the socket server. The camera’s image mode is set to *multiple*, and the trigger mode to *external*. The triggering server is integrated in *TomoScan* and monitors incoming acquire commands during the experiment. For each acquire command received, the server sends a digital trigger to the camera. In this way, the camera is armed only once at the start of a step scan operation.

#### 3.1.3. Data acquisition software

The BEATS data acquisition software system is based on *TomoScan*, an open-source Python module for computed tomography data collection developed at the Advanced Photon Source (APS; Argonne National Laboratory, USA). *TomoScan* consists of a base class implementing beamline-independent functionalities, and of beamline-specific methods implemented in derived classes (Rivers & De Carlo, 2019 ▸). During CT data collection, *TomoScan* controls the operation of various beamline components, including rotary stage, detectors and shutters. Both continuous and step-scan modalities at BEATS are implemented in *TomoScan*. Both cameras of Table 4 ▸ are integrated with *EPICS* (*Experimental Physics and Industrial Control System*; Argonne National Laboratory, USA) through *areaDetector* drivers (Rivers, 2018 ▸). Custom, software-based scan routines were developed to handle the exposure time ranges of each camera as well as frame averaging.

#### 3.1.4. Experimental data streaming

Data streaming refers to the direct transfer of X-ray projections generated by detectors to the STS. The experimental data streaming process handles the creation and storage of experimental files (containing raw data and metadata). A data streaming solution for BEATS was developed with the following requirements:

(i) Adoption of hierarchical data format (HDF5). All BEATS experimental files are created following the scientific data exchange (DXFile) layout (De Carlo *et al.*, 2014 ▸). Each scan file contains metadata describing the acquisition process and experimental conditions.

(ii) Client OS agnostic data processing. The streaming process and writing of experimental files must be compatible with camera drivers running on Linux (*e.g.* FLIR Oryx) as well as Windows (*e.g.* pco.edge) platforms.

(iii) Exploit the full performance of the GPFS centralized storage. GPFS requires its client application to be installed on the client OS (typically Linux) to achieve the maximum read/write performance of the STS.

The streaming of projection frames is implemented using the asynchronous messaging library Zero Message Queuing (ZMQ) (Hintjens, 2013 ▸), allowing reliable communication between distributed systems. With a lightweight design and a variety of programming language bindings, ZMQ facilitates seamless data exchange across networks at low-latency and high throughput. The ZMQ integration with *EPICS* is provided by the *areaDetector* ZMQ plugin (ADZMQ) developed at the Swiss Light Source, Paul Scherrer Institut (Wang, 2021 ▸).

A data writer software (*BEATSH5Writer*) was developed to handle the experiment file creation according to a DXFile layout, and the reception, processing and writing of incoming frames to the HDF5 file. *BEATSH5Writer* can be run as a server continuously or on demand. At the start of an acquisition session, *BEATSH5Writer* is initialized with parameters describing the rotation stage, camera and the scan modality in use (*i.e.* step or continuous scan). Once initialized, *BEATSH5Writer* remains in a listening mode, waiting for a *TomoScan* trigger indicating the start of tomography data collection.

When a new scan is started, *BEATSH5Writer* proceeds to apply SESAME’s naming convention on the experimental data path and associated files. Two beamline-specific XML files (layout and attribute) are used to create HDF5 files in the DXFile format. The XML layout describes the hierarchical structure of the experimental file, while the XML attribute maps each key of the layout file to an active *EPICS* Process Variable (PV).

**Figure 7:**
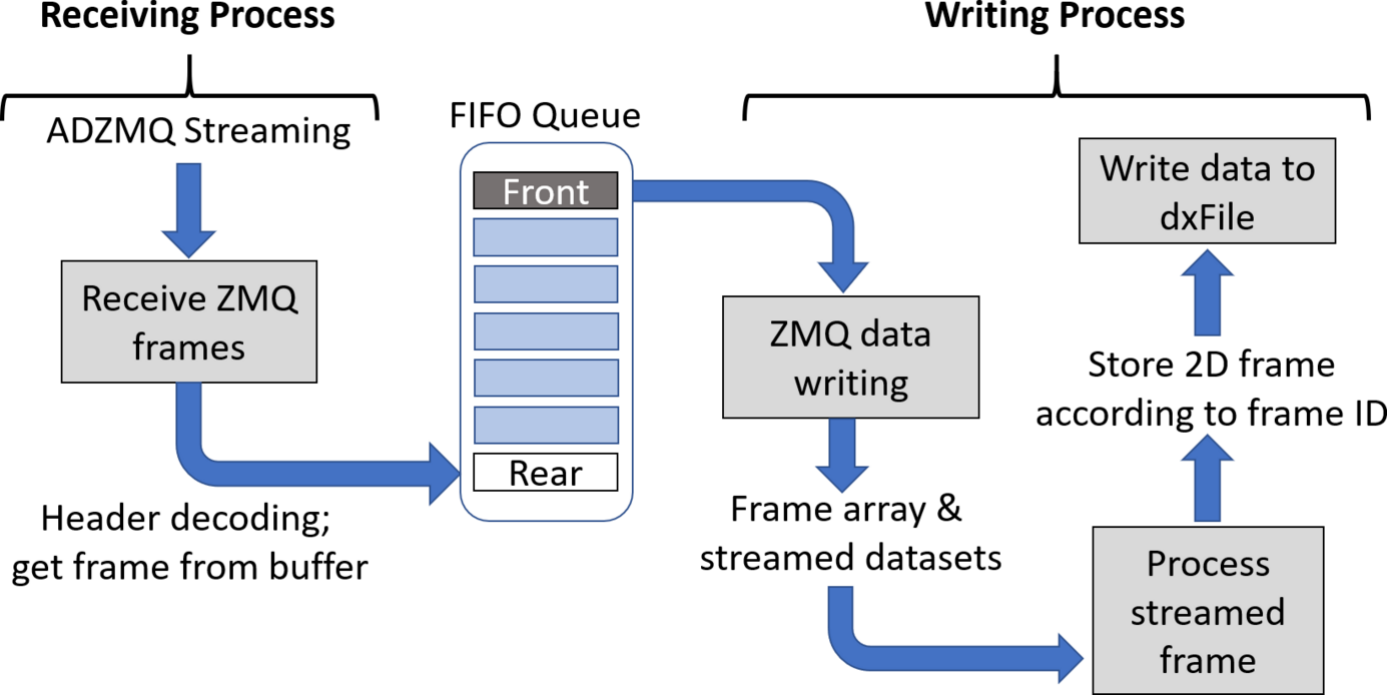
Block diagram illustrating the parallel configuration of receiving and writing processes handled by *BEATSH5Writer*. The FIFO queue is shared by the receiving and writing processes.

Two parallel processes are initialized for receiving the camera frames and for processing and storing them, as shown in Fig. 7 ▸. After launching the processes, *BEATSH5Writer* sends a trigger to *TomoScan* indicating that it is ready to receive the stream of frames. At this point, *TomoScan* initiates the scan procedure.

The receiving process initializes ZMQ context and socket, with the socket set to subscribe to all incoming messages from the publisher (ADZMQ plugin), and then establishes the connection to the socket of the publisher which is on the *areaDetector* driver host. Upon receiving ZMQ messages, the receiving process extracts information from incoming messages including image frames, and stores them at the rear of a first-in first-out (FIFO) queue in the RAM. The contents of the FIFO queue remain available to other parallel processes. The receiving process tracks the number of frames received and stops when it reaches the total number of scan frames provided by *TomoScan*. This is the sum of projections, dark and flat fields. The receiving process also stops when the two following conditions are met: (i) no additional frame is received within a time margin larger than the frame’s exposure time, and (ii) no motor movement is detected while collecting projections. The second condition avoids interrupting the receiving process when as an example the specimen is being inserted or taken out from the field of view for the collection of flat fields.

The writing process retrieves frame information and the image frame itself from the front of the FIFO queue. Each camera frame is received from ADZMQ as a one-dimensional waveform PV and reshaped to a two-dimensional array. The process records the frame identifier and type (*i.e.* dark fields, flat fields and projections), and saves the image to the HDF5 file accordingly. A frame counter is used to monitor and determine the conditions for process completion and termination of the acquisition.

### 3.2. Beamline and experiment control

The control system of BEATS, including personnel and equipment protection layers, was designed and developed at SESAME. Users can control the beamline and experimental parameters through a set of graphical user interfaces (GUIs):

(i) *Beamline synoptic, vacuum and cooling GUI*, used to monitor the status of in-vacuum equipment.

(ii) *Device GUI* allowing the status of motorized in-vacuum equipment such as slits, attenuator and DMM to be modifed.

(iii) *Experiment GUI* for the alignment and configuration of the endstation equipment.

(iv) *Control dashboard* for setting up and monitoring scan procedures. The dashboard allows to select between the different detectors and scan modalities available at the beamline and implements an interlocking mode preventing the simultaneous execution of multiple DAQ instances. Once a detector and scan modality is selected, the scan parameters can be controlled through separate *TomoScan* GUIs (Rivers & De Carlo, 2019 ▸).

(v) *Radiography GUI* for the collection of single radiographs while operating the beamline fast shutter to limit X-ray exposure as described in Section 3.1.1.

(vi) *EPICS SSCAN GUI* used to capture single or multiple data frames (in H5 format) while moving one or more of the beamline positioners through a series of points utilizing the SSCAN functionality. The EPICS scan record utilized by this GUI was developed at the APS (Mooney, 2023 ▸).

(vii) *ImageJ* (Schindelin *et al.*, 2012 ▸) is used in combination with the *ImageJ Channel Access Viewer* included in *EPICS areaDetector* (Rivers, 2018 ▸) to display the detector response during sample alignment and scan setup procedures.

### 3.3. Tomographic reconstruction

Reconstruction of tomographic datasets at BEATS is performed on the two computing nodes of a dedicated hybrid CPU/GPU cluster. Reconstruction jobs are executed using the *TomoPy* (Gürsoy *et al.*, 2014 ▸) and *ASTRA* (Aarle *et al.*, 2016 ▸) Python toolboxes in combination with the *SLURM* workload manager (Yoo *et al.*, 2003 ▸). Each cluster node is equipped with two Intel Xeon Gold 5220R processors, an Nvidia A100 GPU (40 GB memory) and 576 GB of RAM. A GPFS storage system (also dedicated to the beamline) with effective size of 0.51 petabyte is connected to the reconstruction cluster through 100 gigabit per second (Gbps) interconnect. The setup can achieve a sustained read/write data transfer speed of 5 gigabyte per second (GBps) (Iori *et al.*, 2021*b* ▸).

Users have access to predefined reconstruction Python pipelines available as *Jupyter Notebooks* (Granger & Pérez, 2021 ▸). At present, pipelines illustrating CPU (Marone & Stampanoni, 2012 ▸) and GPU reconstruction, automatic centre of rotation detection (Vo *et al.*, 2014 ▸), phase retrieval (Paganin *et al.*, 2002 ▸) and extension of the scan field of view are available, and can be adapted to the needs of each beam time. Alternatively, users who are not familiar with Python can reconstruct their data using *alrecon* (Iori *et al.*, 2024*b* ▸), an open-source *TomoPy* GUI built using *Solara* (Breddels, 2022 ▸). The beamline reconstruction software is hosted on a public GitHub repository (Iori *et al.*, 2024*a* ▸), and is expected to grow hand in hand with the beamline user program.

## 4. Results and beamline performance

The construction of the beamline’s radiation safety hutches and technical infrastructure started in February 2022, followed by the installation of the X-ray source and in-vacuum photon delivery equipment. The first SXCT experiment was performed in May 2023. After commissioning of the beam­line’s DMM optical elements, monochromatic applications have been available since December 2023. The following sections document the instrument’s performance and capabilities.

### 4.1. White beam profile

**Figure 8:**
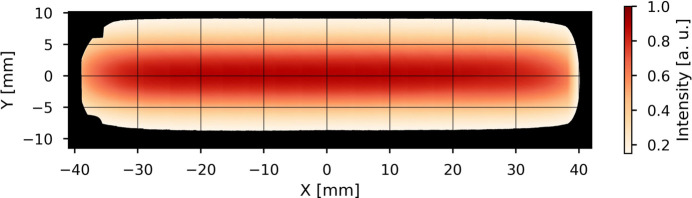
Profile of the filtered white beam at the sample position (43 m from the X-ray photon source) inside the BEATS experimental station. The beam was filtered with 5 mm of glassy carbon and 5 mm of silicon, for a resulting peak X-ray energy of 36 keV. The image is a mosaic of 20 flat-field images collected with 13 µm pixel size and no sample in the detector field of view. When all slits are removed from the beam path, the detected beam edges are shaped by the oval geometry of the copper frame holding the second CVD diamond window. A usable beam size of 75 mm horizontally and 15 mm vertically is available for experiments.

An image of the filtered white beam profile at the sample position (43 m from the X-ray source) is shown in Fig. 8 ▸. The beam size available for experiments is 75 mm × 15 mm, making the endstation of BEATS suited for sample sizes from few millimetres to several centimetres.

### 4.2. Imaging system resolution

**Figure 9:**
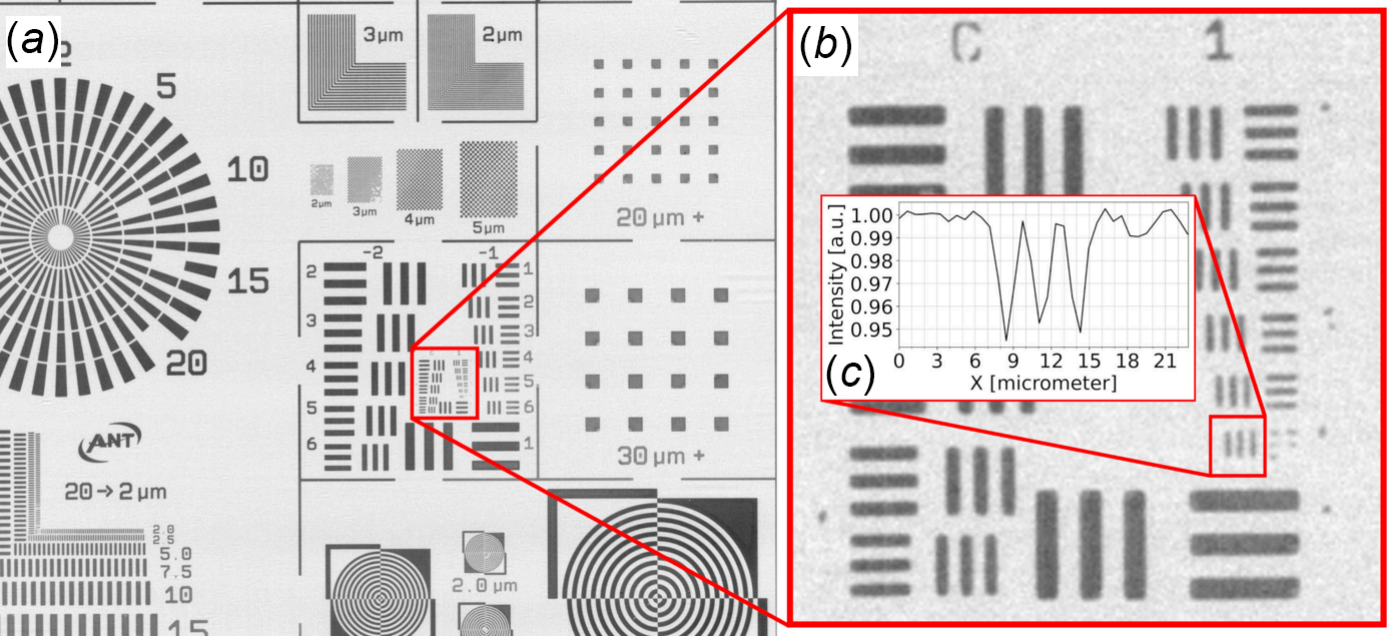
(*a*) Radiograph of the X-ray resolution target (XRCAL-2µm, Applied Nanotools Inc., Canada). (*b*) Detail (enlarged) of (*a*) showing series 0 and 1 of a micro-USAF test pattern. Element 6 of series 1 of the pattern (contoured in red) corresponds to line pairs of 0.357 lp µm^−1^. (*c*) Intensity horizontal line profile through (*b*).

Fig. 9 ▸ shows an X-ray radiograph of a calibration standard target for X-ray imaging systems (XRCAL-2µm, Applied Nanotools Inc., Canada). The image has a pixel size of 0.65 µm and was obtained by setting the X-ray energy to 15 keV using stripe 2 of the BEATS DMM (see Table 2 ▸) and a monochromatic X-ray microscope (detector 3 in Table 3 ▸) with a 15 µm-thick LSO:Tb scintillator (ESRF, France), a 10×/0.30 NA (where NA is the numerical aperture) Plan FLN objective (Olympus, Japan), and a pco.edge 5.5 camera (camera 1 in Table 4 ▸). The target was placed 6 mm in front of the detector scintillator and was visually aligned parallel to it. Exposure time was set to cover 90% of the available dynamic range on the brightest image areas. Twenty images of the target were collected and averaged. Flat and dark fields (average of 20 images each) were collected after removing the target from the field of view, and after closing the beamline shutter, respectively. Each pixel of the target image shown in Fig. 9 ▸(*a*) was normalized as *I*_norm_ = 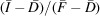, where 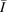, 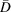 and 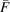 are the averaged pixel values of the target image, dark and flat fields, respectively. In the detail of Fig. 9 ▸(*b*) and line profile of Fig. 9 ▸(*c*), line pairs (lp) of 0.357 lp µm^−1^ are visible, corresponding to a spatial resolution of 2.8 µm.

### 4.3. Data acquisition and reconstruction performance

Different stress tests were conducted to assess the DAQ performance. A fast tomography experiment was performed collecting continuously 10000 projections with 5 ms exposure time of a sample rotating at a speed of 2.44 s for half revolution. Twenty-five 3D datasets were obtained over a time period of approximately 1 min. A stress test of the step-scan data acquisition chain was performed by collecting 10000 projections over a scan duration of approximately 4 h, including acquisition of flat and dark field images before and after the scan. The resulting HDF5 raw data file was 110 GB in size. As expected, the beamline DAQ system was able to handle the loss-less generation of HDF5 files of 100 GB or more at the design throughput of 8.8 Gbps.

The *BEATSH5Writer* presented in Section 3.1.4 could operate both cameras of Table 4 ▸ at the respective maximum frame rate and data throughput. The beamline-specific implementation of *TomoScan* described in Section 3.1.3 could handle sensor exposure times equivalent to or less than the camera frame time, and as low as the values reported in Table 4 ▸.

The performance of BEATS’s tomographic reconstruction HPC facility is demonstrated in Table 5 ▸. The reconstruction speed for a multi-thread CPU implementation of the Gridrec algorithm and for a GPU-accelerated implementation of the filtered back-projection (FBP) are reported for datasets of increasing size. All tests of CT reconstruction performance were scheduled using SLURM on one CPU/GPU node of the beamline’s cluster. Data read and write operations were performed from and to locations on the GPFS STS facility dedicated to the beamline. The module used for reconstruction included the following software versions: *Python* 3.10.10, *TomoPy* 1.11, *astra-toolbox* 2.1.2, *Nvidia CUDA toolkit* 11.3.1. Both CPU- and GPU-based methods were able to reconstruct a dataset of large size (dataset 3 in Table 5 ▸) corresponding to a scan performed by lateral extension of the detector field of view.

## 5. Applications

Images collected during the first months of beamline operation are presented in this section, illustrating experimental possibilities of SXCT at BEATS in four research domains. Reconstruction of 3D volumes was performed with the software presented in Section 3.3. Image analysis and 3D rendering was performed with the Fiji distribution of *ImageJ* (Schindelin *et al.*, 2012 ▸) and *Dragonfly* (Comet Technologies Canada Inc., Montréal, Canada).

### 5.1. Archaeology and cultural heritage

**Figure 10:**
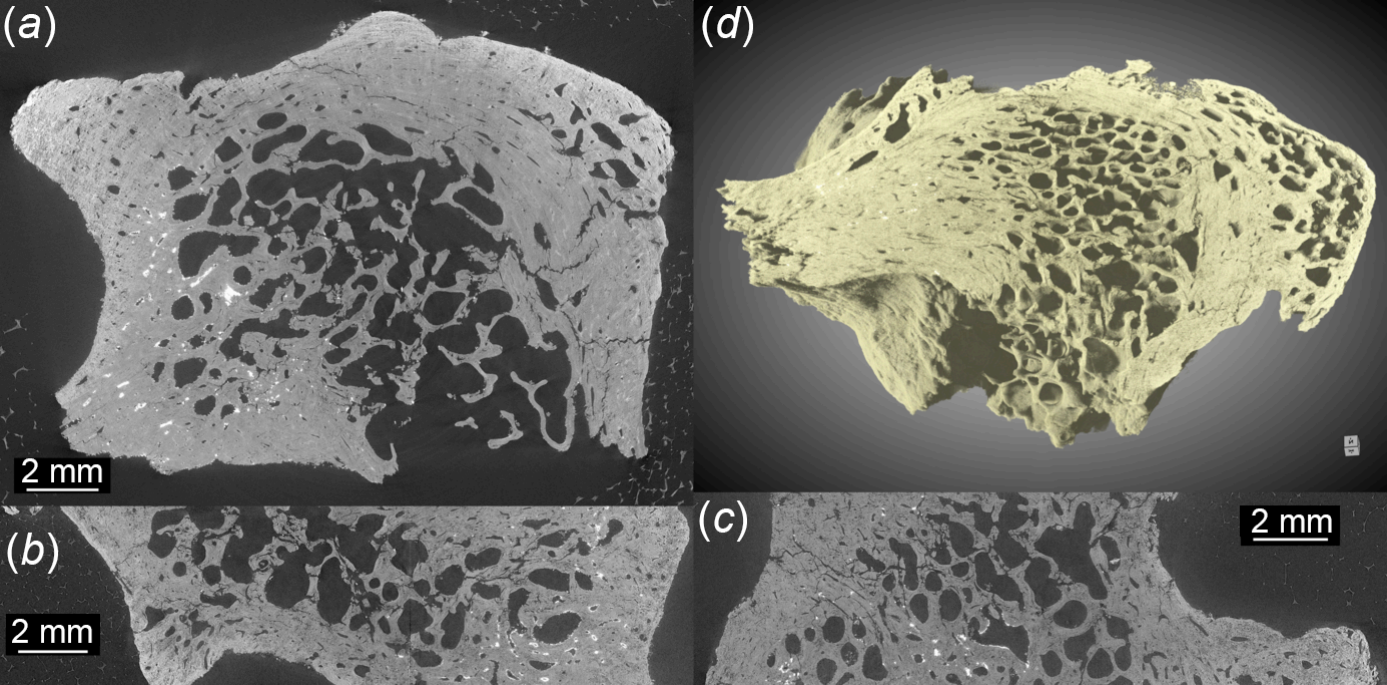
Phase-contrast SXCT scan of an Epipalaeolithic human vertebra from an archaeological excavation carried out in the EMME region. The scan was performed at SESAME BEATS using filtered white beam with peak X-ray energy of 36 keV. Voxel size: 6.5 µm. Number of projections: 8000. Exposure time: 0.7 ms. Scan time: 1.5 min. Transverse (*a*), coronal (*b*) 3D and sagittal (*c*) sections through the reconstructed volume. (*d*) 3D rendering of the scanned region. Thanks to the achievable high 3D resolution and contrast and the possibility of exploiting phase-contrast, SXCT is considered the gold standard for investigations of the morphology and architecture of bone from the millimetre down to the nanometre scale (Maggiano *et al.*, 2016 ▸). Sample courtesy of Dr Kirsi O. Lorentz and Dr Anis Fatima, the Cyprus Institute (Cyprus).

**Figure 11:**
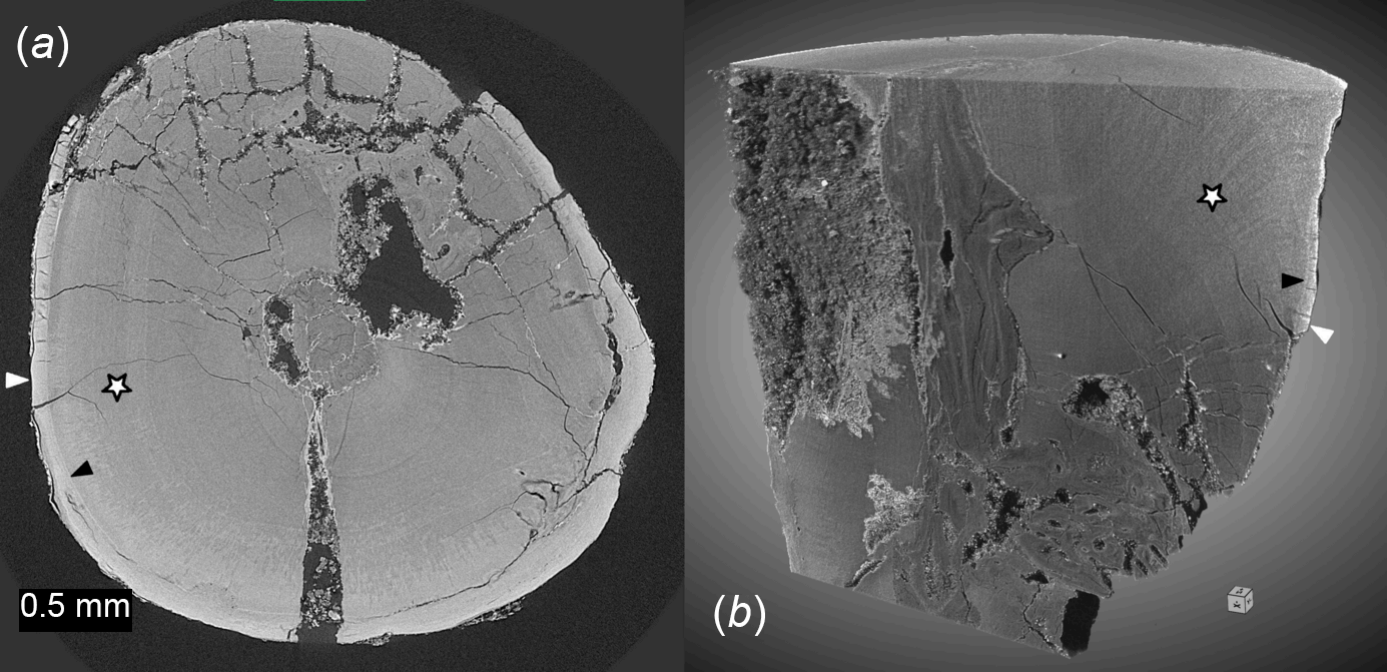
Phase-contrast SXCT scan of human incisor from the Epipalaeolithic period, EMME region. The image was collected at BEATS using a filtered white beam modality with peak X-ray energy of 36 keV and a voxel size of 6.5 µm. Number of projections: 2000. Exposure time: 0.9 ms. Scan time: 30 s. (*a*) Transverse section and (*b*) 3D rendering of the tooth virtually sectioned to expose features of interest. The following anatomical features are labelled: dental cementum (white arrow heads), dentine (stars) and cementodentinal junction (CDJ, black arrow heads). Sample courtesy of Dr Kirsi O. Lorentz and Dr Anis Fatima, the Cyprus Institute (Cyprus).

Possible application of SXCT at BEATS for archaeology and cultural heritage research include the study of archaeological materials such as human, plant or animal remains, and of artefacts made of wood, terracotta, clay, faience, animal bone, antler and teeth (Tafforeau *et al.*, 2006 ▸). Examples of SXCT scans of archaeological human bone and tooth from the Eastern Mediterranean and Middle East (EMME) region performed at BEATS are shown in Figs. 10 ▸ and 11 ▸, respectively. The vertebral segment images of Figs. 10 ▸(*a*)–10(*c*) demonstrate the possibility to quantify the microstructure of cortical and trabecular bone in prehistoric specimens through a non-invasive virtual sectioning approach. Similarly, microscopic anatomical interfaces such as the cementodentinal junction (CDJ; black arrow heads in Fig. 11 ▸) can be analysed from phase-contrast SXCT scans of ancient teeth.

SXCT can be used to examine cultural heritage artefacts such as glass specimens, that are recovered during research excavations after remaining buried in soil for centuries. Different corrosion and alteration processes attributed to the burial environment affect the material properties and appearance of ancient glass samples (Franceschin *et al.*, 2024 ▸). Research on this type of material has the objectives to (*a*) reveal the mechanism of glass degradation induced during burial, (*b*) identify factors (*i.e.* glass composition and burial environment chemistry) that play a role in the alteration of the material, and (*c*) develop conservation approaches and technology that can be applied to preserve cultural heritage objects with anthropological and historical value for future generations. At the same time, the analysis of unique and often delicate artefacts from the past of our civilization requires the use of techniques that are non-invasive. SXCT is non-destructive and does not require sample preparation, representing therefore an unrivalled tool for assessing cultural heritage objects.

**Figure 12:**
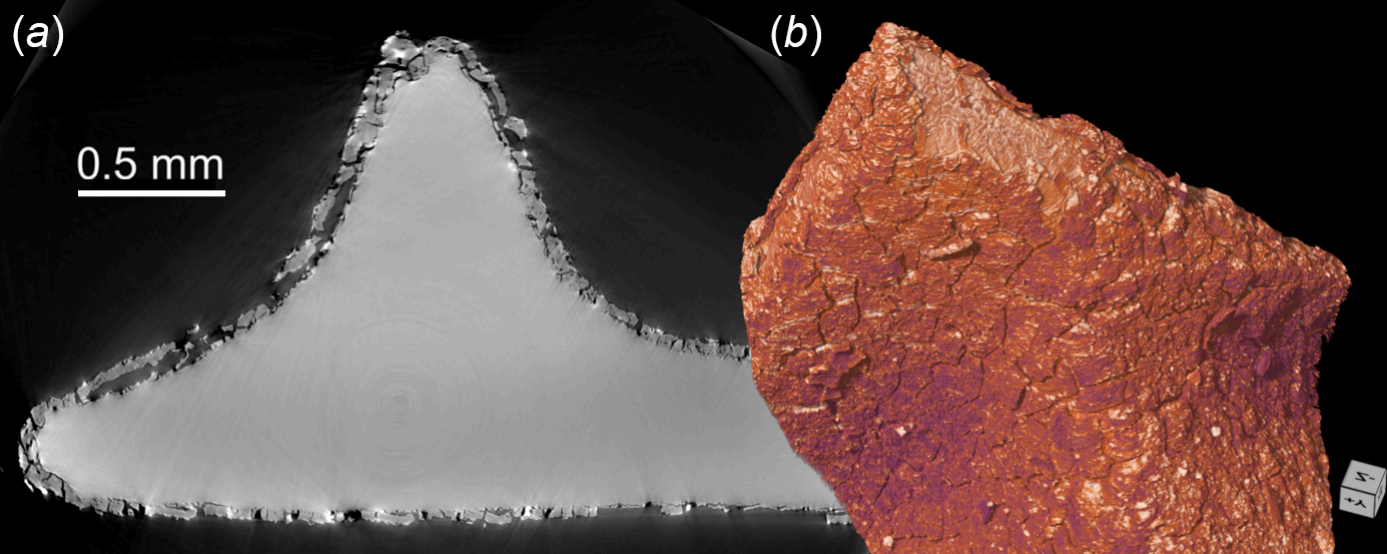
Phase-contrast filtered white beam scan (peak X-ray energy: 25 keV) of historical Roman glass replica subject to artificial degradation. (*a*) Virtual section through the reconstructed volume image: alteration products on the glass surface are distinguishable from the glass bulk due to their different grey scale intensity. (*b*) 3D rendering of the sample. Voxel size: 0.65 µm. Number of projections: 4000. Exposure time: 20 ms. Scan time: 2 min. Sample courtesy of Dr Roberta Zanini and Dr Arianna Traviglia of the IIT Centre for Cultural Heritage Technology (Italy).

SXCT scanning allows the detection and quantification in 3D of morphological details such as microcracks formed due to leaching and chemical attack on the glass surface. Fig. 12 ▸ shows the results of a phase-contrast SXCT scan performed at BEATS of a Roman glass replica subject to artificial ageing as described by Zanini *et al.* (2023 ▸). The use of partially coherent synchrotron radiation enables phase-contrast image formation to be exploited and the contrast between glass and altered surface layers to be enhanced, revealing the extent and morphology of glass alteration and cracking. Such information can be used to model and predict glass failure. Given the rich archaeological environment surrounding SESAME, SXCT at BEATS can be a valuable tool for cultural heritage researchers, historians and conservators working on rare glass objects (Barfod *et al.*, 2018 ▸).

### 5.2. Life sciences

**Figure 13:**
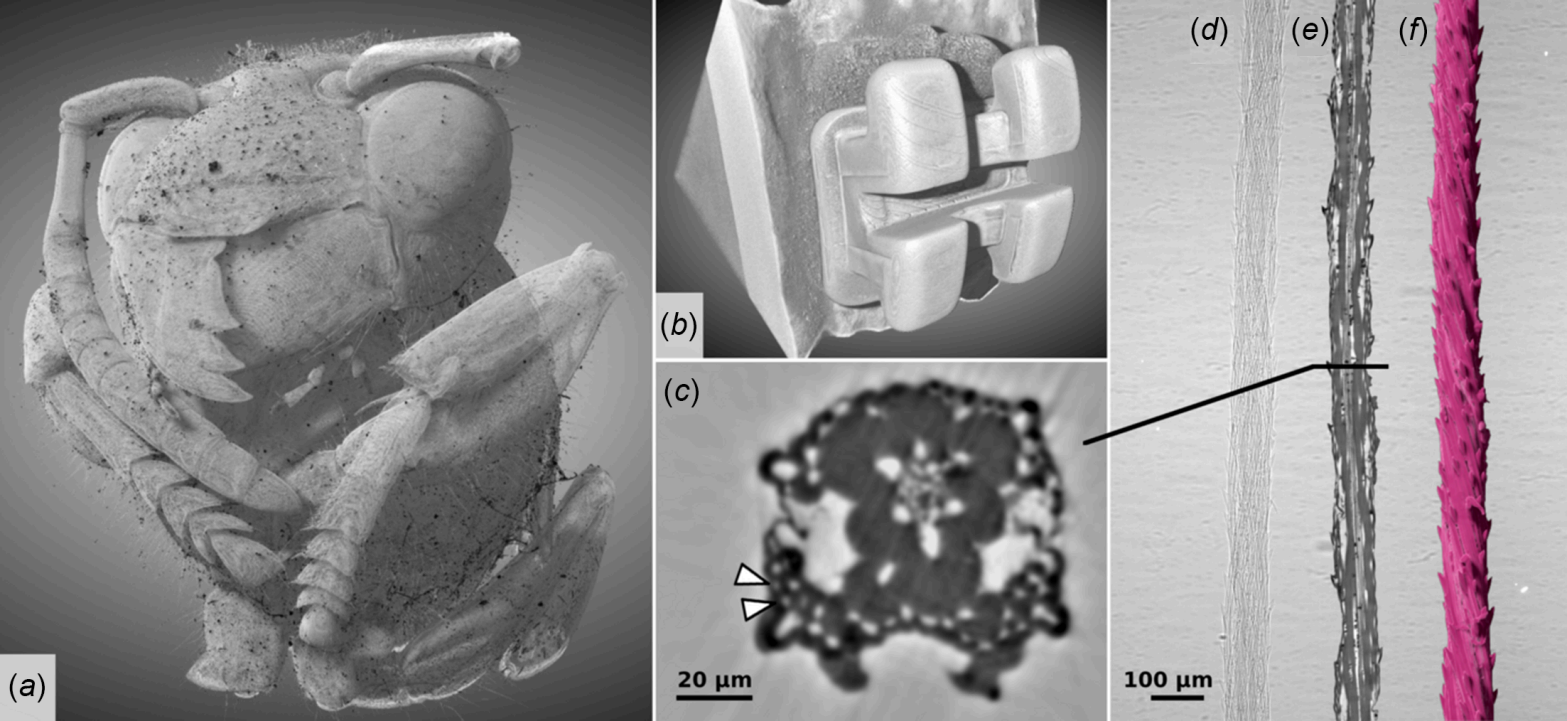
SXCT images of life science samples from SESAME BEATS. (*a*) 3D rendering from phase-contrast reconstruction of a German wasp (*Vespula germanica*). Filtered white beam (peak X-ray energy: 25 keV). Voxel size: 3.1 µm. Number of projections: 2000. Exposure time: 20 ms. Scan time: 55 s. (*b*) 3D visualization of ceramic orthodontic bracket bonded to bovine tooth model. Filtered white beam (peak X-ray energy: 36 keV). Voxel size: 4.5 µm. Number of projections: 4000. Exposure time: 8.4 ms. Scan time: 2 min. (*c*) Transverse section, (*d*) radiograph, (*e*) longitudinal section and (*f*) 3D volume rendering of a thin grass fibre (diameter 90 µm approximately). The scan was performed with filtered white beam at a peak X-ray energy of 16 keV and a voxel size of 0.65 µm. Number of projections: 1000. Exposure time: 30 ms. Scan time: 40 s. Despite low X-ray absorption, high contrast and anatomical resolution are achieved in the reconstructed images [(*c*) and (*e*)] thanks to a phase-retrieval step. Microvessels with a diameter of approximately 2.5 µm are highlighted with arrow heads in (*c*). Sample in (*b*) courtesy of Dr Petra Koch from Charité – Universitätsmedizin Berlin (Germany). The scans shown in (*c*), (*d*), (*e*) and (*f*) are courtesy of Dr Marieh Al-Handawi and Professor Panče Naumov from New York University Abu Dhabi (UAE).

X-ray CT allows biological samples to be inspected and analysed at micrometre and sub-micrometre resolution in 3D and without damaging the tissue. This is paramount to understanding the structural–functional relationships in living tissues and organs as well as the effect of medications and external agents on these (Rawson *et al.*, 2020 ▸). Applications of SXCT in life sciences are shown in Fig. 13 ▸. Fig. 13 ▸(*a*) shows the 3D rendering obtained from a phase-contrast SXCT scan of *Vespula germanica*. Phase-contrast modalities can boost the contrast of images of otherwise low-absorbing anatomical structures, making SXCT a valuable tool for research in entomology. Scans can be applied for the observation and description of modern or fossil insect species (Bukejs *et al.*, 2019 ▸). Bone and dentistry research are examples of widespread application of SXCT in biomedical research. A volume rendering from a SXCT scan of a ceramic dental bracket applied to a bovine tooth is shown in Fig. 13 ▸(*b*). Dental brackets are used in orthodontic treatment to help correct the alignment of teeth and jaws, and are bonded to the surface of the tooth crown using special dental adhesives. The resistance of orthodontic bonding and possible enamel damage provoked by appliance removal can be studied visualizing the architecture and microstructure of the tooth-bracket complex with SXCT. Further examples in dentistry research include the optimization of root canal treatment (Prates Soares *et al.*, 2020 ▸), and the quantitative analysis of the enamel morphology and density profile during demineralization of the tooth surface (Lautensack *et al.*, 2013 ▸).

Phase-contrast SXCT can be applied for plant tissue characterization, on both fresh samples and archaeological plant remains. On modern species, SXCT can be utilized to study plant anatomy, root architecture and soil interaction (Moran *et al.*, 2000 ▸), as well as water movement and uptake. SXCT can also help in identifying the species of plant remains from archaeological contexts (Calo *et al.*, 2019 ▸). Figs. 13 ▸(*c*)–13(*f*) present a high-resolution, phase-contrast SXCT scan of a thin plant fibre performed at SESAME BEATS with scan time below 1 min. The visibility of microvessels with diameter as low as 2.5 µm [white arrow heads in Fig. 13 ▸(*c*)] is in agreement with the measurement of the imaging system resolution presented in Section 4.2.

### 5.3. Material science and engineering

**Figure 14:**
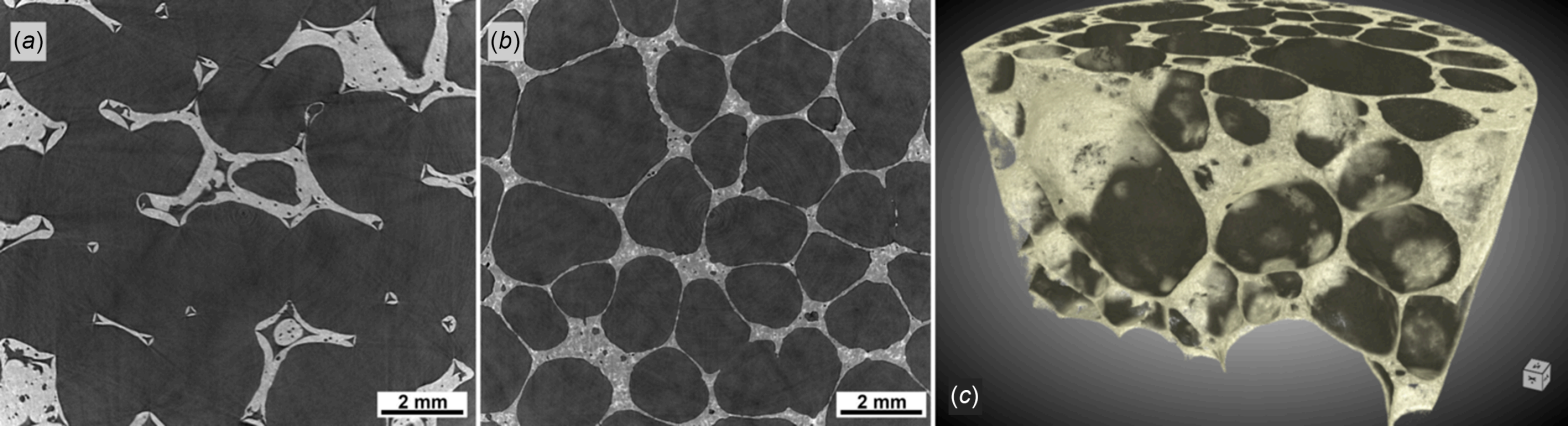
(*a*) Virtual section through an open-cell ceramic sponge. (*b*) Virtual section and (*c*) 3D rendering of an aluminium alloy closed-cell foam sample (AlSi6Cu4). Both scans were performed with filtered white beam (peak X-ray energy: 36 keV) and a voxel size of 3.1 µm. Number of projections: 4000. Exposure time: 11 ms. Scan time: 1 min. Structural defects and the distribution of imperfections can be studied with micrometre resolution over large portions of the material, without the need for sample preparation. Foaming processes can be tracked *in situ* by hard synchrotron X-ray radiography as demonstrated at beamline ID19 of the ESRF (Mukherjee *et al.*, 2017 ▸).

SXCT is widely used to study and develop light and composite materials for construction and transport engineering, as well as for energy materials research (Maire & Withers, 2014 ▸; Banhart, 2001 ▸). Engineering of light materials is an essential step in the design of modern transportation systems and construction materials, with weight reduction in packaging and vehicles being a leading factor in the reduction of energy consumption. Fig. 14 ▸ shows preliminary filtered white beam scans of closed and open foam cellular materials obtained at BEATS. A cross section through an open-cell ceramic sponge is shown in Fig. 14 ▸(*a*). Figs. 14 ▸(*b*) and 14(*c*) show a section and 3D rendering of a reconstructed volume of a closed-cell AlSi_6_Cu_4_ foam sample. The scan was performed using filtered white beam (mean energy: 30 keV), detector 2 in 1× magnification (see Table 3 ▸ for details), and a pco.edge 5.5 camera, giving a scan voxelsize of 6.5 µm. The time for a 3D scan was less than 1 min. AlSi_6_Cu_4_ foam is a lightweight cellular material with possible applications in various engineering fields due to its unique combination of low weight, considerable mechanical strength, thermal and acoustic/vibration management. Aluminium and other metals foams are applied in aerospace and automotive engineering, in heat exchangers and acoustic dampers or insulators, as well as for lightweight biomedical implant or prosthesis fabrication. Classical challenges for metal foams are the standardization of a homogeneous pore structure as well as its reproducibility, with the mechanical properties of the foamed part being affected by the non-uniform distribution of cell size, by the lack of connectivity in cell walls, and by the presence of microporosity (Jeon & Asahina, 2005 ▸). Different *in situ* and *ex situ* studies can be performed at BEATS, which are useful for the optimization of foam properties and to gain knowledge about the casting process.

**Figure 15:**
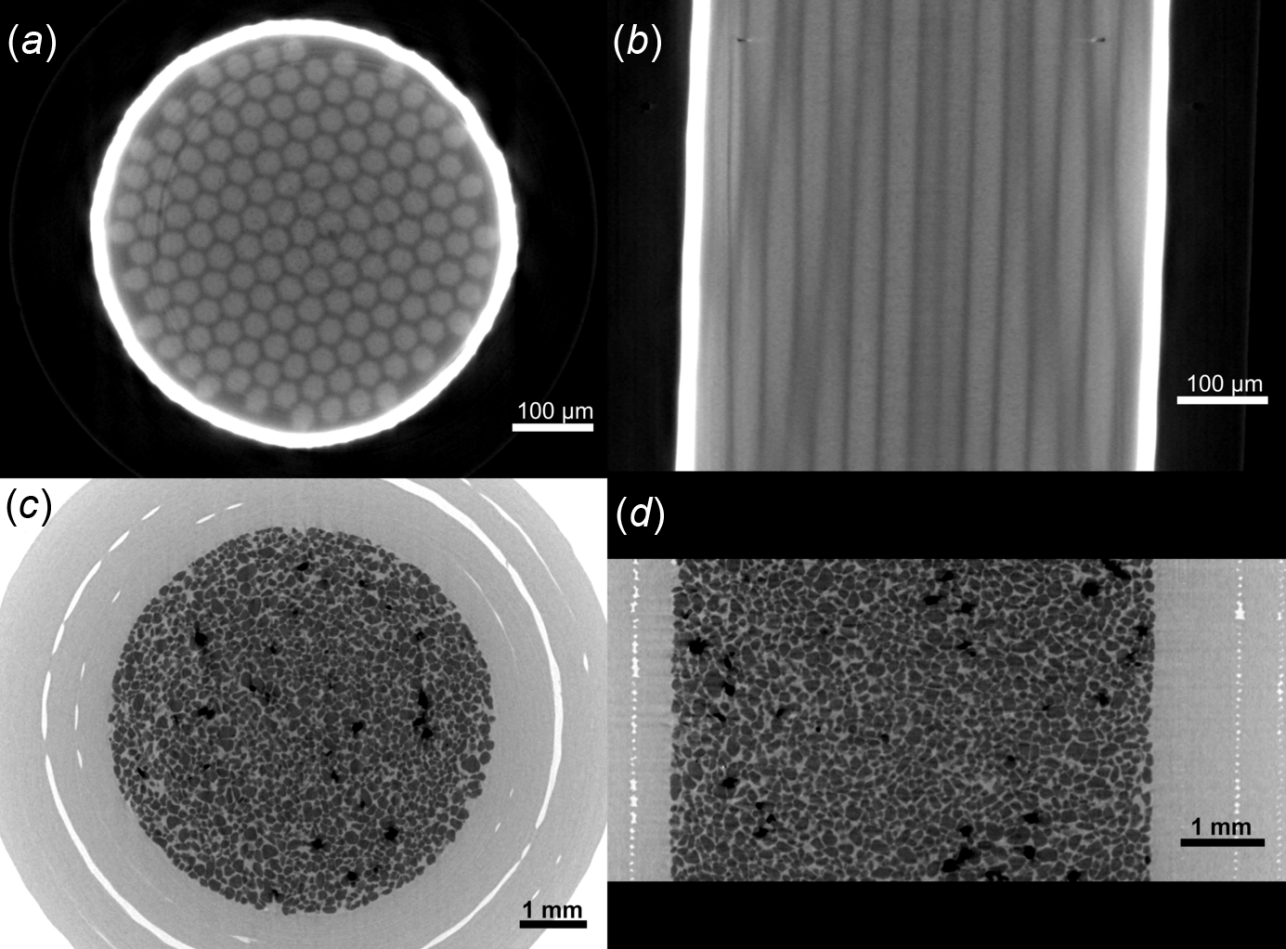
White beam SXCT applications in material and earth sciences at SESAME BEATS. (*a*) Transverse and (*b*) longitudinal sections through the reconstruction of an Nb_3_Sn superconducting wire. Scan performed with filtered white beam with peak X-ray energy of 69 keV. 3D image voxel size: 1.3 µm. Number of projections: 10000. Exposure time: 2 s. Scan time: 6 h. The sample was part of a measurement campaign performed at beamline ID19 of the ESRF (France) (Barth *et al.*, 2018 ▸) and is courtesy of Dr Christian Barth, Dr Tommaso Bagni and Professor Carmine Senatore from the University of Geneva (Switzerland). (*c*) Transverse and (*d*) longitudinal sections through the reconstruction of a wetting experiment on a quartz sand sample (F-75 silica). Scan performed with filtered white beam with peak energy of 36 keV. 3D image voxel size: 6.5 µm. Number of projections: 1000. Exposure time: 17 ms. Scan time: 20 s. Sample courtesy of Dr Jamal Hannun and Professor Riyadh Al-Raoush from Qatar University.

Figs. 15 ▸(*a*) and 15(*b*) demonstrate the application of SXCT at SESAME BEATS for the imaging of a Nb_3_Sn superconducting wire sample. Wires of niobium–tin compounds exhibit unparalleled superconducting properties at high temperatures, enabling the creation of powerful magnetic fields that are crucial in various scientific and industrial applications. Due to their exceptional performance, Nb_3_Sn superconducting wires are at the forefront of cutting-edge power transmission, high-energy physics and magnetic resonance imaging technology. Several techniques have been proposed for the fabrication of Nb_3_Sn wires. In Fig. 15 ▸(*a*), single thin Nb filaments of a multifilamentary Nb_3_Sn composite wire are visible. The Sn-rich core is separated by the surrounding high-purity Cu layer by a single Ta diffusion barrier [bright envelope in Fig. 15 ▸(*a*)]. Nb_3_Sn wires such as the one shown in Figs. 15 ▸(*a*) and 15(*b*) were the object of an experiment campaign at beamline ID19 of the ESRF (France). This was designed to quantify the presence and morphology of voids (Bagni *et al.*, 2021 ▸) forming within the wire during fabrication and heat-treatment processes, and affecting the performance of the superconductor. Due to the small size and high density of the material, the scanning of this type of samples requires a unique combination of high energy and high spatial resolution.

### 5.4. Earth sciences

SXCT at SESAME BEATS can provide an important tool for research in geology, environmental sciences, agriculture and plant research. Example applications include the characterization of soil microstructure, *i.e.* how this is influenced by agricultural techniques (Cooper *et al.*, 2021 ▸), and how it interacts with plant roots (Moran *et al.*, 2000 ▸). Phase-contrast SXCT of geological samples can inform the analysis of pores or grain size, shape and distribution, and the simulation of fluid flow through rocks or sediments (Kakouie *et al.*, 2021 ▸). Images from a demonstration fast scan performed with filtered white beam at SESAME BEATS during the wetting of quartz sand are shown in Figs. 15 ▸(*c*) and 15(*d*). A microscopic 3D visualization of the interaction between fluid and sediments during clogging or wetting can be used to validate and improve large-scale models of fluid-soil dynamics (Jarrar *et al.*, 2021 ▸). Understanding soil permeability and subsurface water mechanisms is a crucial step for groundwater protection, an urgent task for the Middle East, where several countries are already experiencing extremely high water stress (Kuzma *et al.*, 2023 ▸).

## 6. Summary and outlook

In this article we have presented the synchrotron X-ray tomography beamline BEATS of SESAME. The beamline was installed between February 2022 and May 2023, when the first X-ray tomograms were collected in its experimental station. ID10-BEATS was officially inaugurated on 6 June 2023, and has been open for SESAME users since January 2024, as the fifth beamline of the facility going online. Thanks to a wavelength shifter insertion device and double-multilayer monochromator optics, the intensity and energy spectrum of the available X-ray beam can be tuned to the required characteristics. The radiography and microtomography endstation of BEATS uses a high-precision sample manipulator and indirect X-ray detectors based on scintillating crystals, visible-light optics and sCMOS cameras. A broad range of image magnifications is available, allowing the scan of samples of various size and geometry. The beamline’s data acquisition and reconstruction system implements scan modalities for different experimental conditions, efficient streaming of tomograms to a centralized storage system, and fast CT reconstruction on a dedicated CPU/GPU cluster, allowing users to perform high-throughput experiments. The following upgrades are being prepared to expand the equipment portfolio of the beamline:

(i) Sample manipulator for high payloads. A heavy-duty, five-axis sample manipulator based on air-bearing technology and assembled on a granite stage independent of the detector table is currently being manufactured. This will allow sample payloads up to 50 kg, and rotation speed up to 60 rpm. The system will also extend the maximum available propagation distance between sample and detector to approximately 6 m.

(ii) Sample environment for mechanical testing. A 1000 N compression-tensile mechanical testing stage specifically designed for X-ray tomography will be available for *in situ* experiments under displacement control. Objects up to 22 mm in diameter and 33 mm in length can be mounted for testing. Load transfer between the stage components is accomplished with a circular window in polycarbonate or aluminium, depending on the scan X-ray energy. The possibility to vary the mechanical load applied on a sample while imaging its interior in 3D has wide potential for application in the fields of materials science and engineering, biomedical research, and more. The high photon flux available in white beam modality allows fast scanning and time-resolved analyses while mechanical tests are performed.

(iii) Sample environment for temperature control. A furnace for *in situ*, temperature-controlled experiments based on an induction heating system will be also available. The sample environment will allow temperatures from ambient up to 1200°C. Samples and materials that are not electrically conductive can be studied by sliding them inside apposite metal crucibles.

Our report describes the first use of SXCT in the Middle East and Eastern Mediterrenean region, with spatial resolution higher than 2.8 µm (as verified with a resolution test pattern), scan time as low as a few seconds, and the possibility to reconstruct in a widely non-destructive manner the local X-ray attenuation coefficient or phase shift of specimens. The research potential released by the new beamline of SESAME was illustrated with examples of SXCT imaging of materials and samples from archaeology, cultural heritage, materials, life and earth sciences. The possibility to characterize the internal microstructure of specimens at high spatial and temporal resolution, and without sectioning or damaging the object under investigation is a key step for an exhaustive understanding of materials, artefacts and organisms from the past and present of our civilization.

## Supplementary Material

Synchrotron X-ray Computed Tomography scan of a wasp: https://doi.org/10.5281/zenodo.10075277

## Figures and Tables

**Table 1:** BEATS X-ray source parameters

Type	Three-pole wiggler
Minimum gap	11.15 mm
Peak magnetic field	2.9 T
Critical energy	12.0 keV
Maximum emitted power	870 W
Photon source size (FWHM)	1.9 mm × 32.7 µm (H × V)
Magnetic length	412 mm
Manufacturer	Kyma SpA (Italy)

**Table:** Measured metrology and reflectivity values are reported. Two reflections are considered for reflectivity and energy bandwidth values.

	Units	Value
General specifications		
Distance from source (multilayer 1)	m	16.1
Maximum incident power (on multilayer 1)	W	132.7
Beam offset (variable)	mm	4.0–20.0
Substrate dimensions (L × W × H)	mm	500 × 70 × 60
Clear aperture (L × W)	mm	480 × 55
Substrate longitudinal slope error	µrad	0.27
Substrate surface roughness (r.m.s.)	Å	2.9
Monochromator manufacturer	Strumenti Scientifici CINEL (Vigonza, Italy)	

**Table:** 

	Unit	Multilayer 1	Multilayer 2
Performance and stability			
Cooling type		Ga–In alloy bath and water cooling	None
Pitch resolution (open loop)	µrad	0.453	0.440†
Pitch repeatability (open loop, bi-directional)	µrad	1.798	1.374‡
Natural frequencies (largest amplitude modes)	Hz	82; 90	87; 96
20 min vibration stability (r.m.s.)	nrad	22	19

**Table:** 

	Units	Stripe 1	Stripe 2
Multilayer coatings			
Coating		[W/B_4_C]_100_	[Ru/B_4_C]_65_
*d*-spacing	nm	2.499	4.030
*d*-spacing longitudinal gradient	%	3.3 (over 480 mm)	3.4 (over 480 mm)
Number of bilayers		100	65
Energy	keV	18–60	7–25
d*E*/*E*	%	1.6	2.4
θ (Bragg angle)	°	0.243–0.808	0.367–1.311
Filling factor (Γ)		0.50	0.47
Reflectivity (*R*^2^)	%	0.80 (@ 50 keV)	0.81 (@ 22 keV)
Multilayer deposition and characterization	ESRF Multilayer Laboratory (Grenoble, France)		

† <20 nrad with piezoelectric actuator.

‡ <4.33 nrad with piezoelectric actuator.

**Table 3:** Indirect X-ray detector systems available for experiments Object pixel sizes are provided for the range of available magnifications, and considering a pco.edge 5.5 camera (Camera 1 in Table 4 ▸).

	Detector 1	Detector 2	Detector 3
Type	Tandem lens macro	Microscope	Microscope
Lens type	Hasselblad H system	Mitutoyo M Plan Apo (radiation hardened)	Olympus PLAPO/UPLAPO
White beam compatible	Yes	Yes	No
Magnification	0.5× to 2×	5× to 10×	4× to 20×
Pixel size	13.6–3.1 µm	1.30–0.65 µm	0.65–0.33 µm
Maximum field of view (horizontal)	33.28 mm	3.33 mm	4.16 mm
Scintillator	LuAG:Ce	GGG:Eu; LSO:Tb	
Manufacturer	ESRF (France)	Optique Peter (France)	

**Table 4:** Specifications of the scientific cameras available at the BEATS imaging endstation Both cameras can be used in combination with all detectors of Table 3 ▸.

	Units	Camera 1	Camera 2
Sensor type		sCMOS – Mono	CMOS – Mono
Manufacturer		PCO AG (now Excelitas Technologies, USA)	Teledyne FLIR (USA)
Model		pco.edge 5.5 CLHS	Oryx 10GigE
Resolution		5.5 MP	7.1 MP
Sensor size	pixels	2560 × 2160	3208 × 2200
Pixel size	µm	6.5	4.5
MaxIMUM frame rate (full frame)	frames s^−1^	100	112†
Shutter		Rolling / global	Global
Exposure time		500 µs to 2 s	10 µs to 30 s
Bit-depth	bit	16	8 / 16
ADC	bit	16	8 / 10 / 12
Full-well capacity	e^−^	30000	24500
Dynamic range	dB	88.6	71.7
*EPICS areaDetector* driver		ADPcoWin	ADSpinnaker

† With 8-bit ADC.

**Table 5:** Reconstruction time for datasets of different size collected at BEATS Reconstructions were performed using the *TomoPy* Gridrec method and the CUDA filtered back projection (FBP) implementation of the *ASTRA toolbox* on one CPU/GPU node of the beamline’s hybrid reconstruction cluster (see Section 3.3 for cluster specifications). The stack height was 1000 pixels for all datasets. The time required by the CT reconstruction step is reported in seconds. The time including all steps of the reconstruction pipeline (HDF5 data read from GPFS storage, CPU/GPU computation, and writing of 32bit reconstructed tiff slices) is reported in parentheses.

Test dataset	Dataset 1	Dataset 2	Dataset 3
Sinogram shape	1376 × 1000 × 1000	2560 × 1000 × 1000	4371 × 1000 × 2000
Raw data size (16-bit)	6.0 GB	28.2 GB	42.3 GB
Reconstruction shape	1376 × 1376 × 1000	2560 × 2560 × 1000	4371 × 4371 × 1000
Reconstruction size (32-bit)	7.6 GB	26.2 GB	76.4 GB
Gridrec	5.6 s (50.5 s)	18.8 s (117.5 s)	88.5 s (268.4 s)
FBP CUDA	51.1 s (95.7 s)	69.2 s (169.3 s)	249.6 s (421.3 s)
